# Increased *E2F1* mRNA and miR-17-5p Expression Is Correlated to Invasiveness and Proliferation of Pituitary Neuroendocrine Tumours

**DOI:** 10.3390/diagnostics10040227

**Published:** 2020-04-16

**Authors:** Araceli García-Martínez, Beatriz López-Muñoz, Carmen Fajardo, Rosa Cámara, Cristina Lamas, Sandra Silva-Ortega, Ignacio Aranda, Antonio Picó

**Affiliations:** 1 Research Laboratory, Hospital General Universitario de Alicante-Institute for Health and Biomedical Research (ISABIAL), 03010 Alicante, Spain; araceli86gm@gmail.com; 2 Department of Endocrinology & Nutrition, Hospital General Universitario de Alicante -ISABIAL, 03010 Alicante, Spain; bealopezmz@gmail.com; 3 Department of Endocrinology and Nutrition, Hospital La Ribera, Alzira, 46600 Valencia, Spain; fajardo_carmon@gva.es; 4 Department of Endocrinology & Nutrition, Hospital Universitario y Politécnico La Fe, 46026 Valencia, Spain; rosacamaragomez@gmail.com; 5 Department of Endocrinology & Nutrition, Hospital General Universitario de Albacete, 02006 Albacete, Spain; clamaso@sescam.jccm.es; 6 Department of Pathology, Hospital General Universitario de Alicante -ISABIAL, 03010 Alicante, Spain; ssilvaortega@yahoo.es (S.S.-O.); ignaranda@gmail.com (I.A.); 7 Department of Endocrinology & Nutrition, Hospital General Universitario de Alicante, Miguel Hernández University, 03010 Alicante, Spain

**Keywords:** miR-17~92, pituitary neuroendocrine tumours, *E2F1*, microRNAs

## Abstract

miR-17-5p and *E2F1* have been described as deregulated in cancer, but they have scarcely been studied in pituitary neuroendocrine tumours (PitNETs). This study evaluates the relationship of *E2F1* and miR-17-5p with the invasiveness and proliferation of PitNETs. In this cross-sectional descriptive study, we evaluated the expression of *E2F1*, *MYC*, and miR-17-5p by quantitative real time PCR analysis in 60 PitNETs: 29 gonadotroph (GT), 15 functioning somatotroph (ST), and 16 corticotroph (CT) tumours, of which 8 were silent (sCT). The clinical data were collected from the Spanish Molecular Register of Pituitary Adenomas (REMAH) database. We defined invasiveness according to the Knosp classification and proliferation according to a molecular expression of Ki-67 ≥ 2.59. *E2F1* was more expressed in invasive than in non-invasive tumours in the whole series (*p* = 0.004) and in STs (*p* = 0.01). In addition, it was overexpressed in the silent subtypes (GTs and sCTs; all macroadenomas) and normoexpressed in the functioning ones (fCTs and STs; some microadenomas). miR-17-5p was more expressed in proliferative than in non-proliferative tumours (*p* = 0.041) in the whole series but not by subtypes. Conclusions: Our study suggests that in PitNETs, *E2F1* could be a good biomarker of invasiveness, and miR-17-5p of proliferation, helping the clinical management of these tumours.

## 1. Introduction

One of the hallmarks of cancer in humans is the deregulation of E2F transcriptional activity through the alteration of the p16INK4a-cyclinD1-retinoblastoma protein (Rb) pathway. E2F is a family of transcription factors that controls the expression of important genes for the progression of the cell cycle, apoptosis, DNA repair, and cellular differentiation. *E2F1* is a transcription factor belonging to the E2F family that has the ability to induce two seemingly contradictory processes. Its activation can stimulate the growth and the proliferation of the cells, but it also participates in the apoptotic process, protecting against tumourigenesis [1,2,3]. *E2F1* is widely involved in tumorigenesis [1,3,4]. In pituitary neuroendocrine tumours (PitNETs), its participation in the growth and proliferation of the tumours has been related through its relationship with other factors such as *HMGA1* [5] or *PTTG1* [6].

*E2F1* is under the transcriptional regulation of *MYC* [7], and it has also been shown that some miRNAs have a great influence on its activity [4,8,9,10]. Moreover, miRNAs have also been involved in the tumourigenesis process of PitNETs [11]. Indeed, Bottoni et al. identified 30 miRNAs that are differentially expressed in normal and adenomatous pituitary tissues [12]. Pri-miR-17-92 is a primary miRNA that gives rise to the mature miRNAs 17, 18a, 19a, 20a, 19b, and 92a when processed. This polycistronic complex is under the regulation of *MYC* [13] and factors of the E2F1 family [14]. However, it has never been studied in PitNETs.

Therefore, the aim of this study was to assess the relationship between the expression of *E2F1* and the behaviour of PitNETs and its regulation by *MYC* and the miR-17-92 cluster.

## 2. Materials and Methods

This study complies with the Declaration of Helsinki and was approved by the local ethics committee (2012/08 of 27 June 2012). All patients signed an informed consent.

### 2.1. Samples

We selected 60 PitNETs from the biobank pituitary collection of the Alicante General University Hospital and 9 normal pituitary glands obtained from autopsies performed at the same centre. All specimens were preserved within 24 h to 48 h after surgery at 4 °C in RNAlater solution and frozen at −80 °C.

The Spanish Molecular Registry of Pituitary Adenomas (REMAH) network initiative allowed us to identify the molecular subtypes of PitNETs [15,16,17]. Namely, we identified the molecular subtypes of PitNETs based on the gene expression of pituitary hormones and some receptors (*POMC*, *CRHR1*, *AVPR1B*, *PRL*, *TSH*, *GH*, *FSH*, and *LH*). After that, we chose the overall sample of this study, composed of 29 GTs (including 23 follicle-stimulating-hormone-secreting tumours (FSHoma), 1 luteinising-hormone-secreting-tumour (LHoma) and 5 mixed-gonadotropin-secreting -tumours), 8 sCTs (not expressing Cushing’s syndrome), 8 fCTs (expressing Cushing’s syndrome), and 15 STs (expressing acromegaly syndrome).

### 2.2. Clinical, Radiological, and Pathological Data of the Patients

Patients’ gender, age, and clinical and radiological data were retrieved anonymously. Clinical information included: endocrine or neuro-ophthalmological symptoms and signs, and biochemical evaluation of the pituitary function.

Two investigators independently reviewed the magnetic resonance images (MRIs) of selected patients. All exams where performed on a 1.5 Tesla magnet Philips Intera (Philips, Amsterdam, Netherlands) according to a predefined protocol including contrast enhanced dynamic and delayed image acquisitions. From these images, tumours were classified according to invasiveness and maximum tumour diameter (MTD). In terms of invasiveness, tumours were classified as invasive (Knosp grades III–IV) or non-invasive (Knosp grades I–II) [18].

Pathological data included the measurement of the relative gene expression of the Ki-67, because it was not possible to centralise its immunohistochemical (IHC) evaluation. To define a Ki-67 gene expression fold change (FC) equivalent to the 3% IHC cutoff, we performed a receiver operator curve analysis (ROC analysis). As a result, we found that 2.59 FC Ki-67 gene expression provided the highest sensitivity (85.5%) and specificity (77.6%). Therefore, tumours were classified as proliferative (Ki-67 ≥ 2.59) or non-proliferative (Ki-67 < 2.59).

According to the clinicopathological classification proposed by Trouillas et al. [19,20], we defined three grades: 1. non-invasive tumours, regardless of the Ki-67 index (value from 0.081 to 11.631; 6 tumours with ki-67 ≥ 2.59), 2. invasive and non-proliferative tumours with Ki-67 index < 2.59, and 3. invasive and proliferative tumours with Ki-67 index ≥ 2.59. Grade 1 corresponds to grades 1a and 1b of the Trouillas’ classification; grades 2 and 3 are similar to grade 2a and grade 2b respectively [19].

In all tumours, we quantified the gene expression of *E2F1*, *MYC*, pri-miR-17~92, miR-17-5p, and miR-20a.

### 2.3. Nucleic Acid Extraction

All experiments were performed equally on problem cases and normal pituitary glands. Tissue fragments, obtained from representative areas of the tumour, were efficiently disrupted, lysed, and homogenised using the TissueLyser (Qiagen, Hilden, Germany) and the corresponding buffer. Genomic DNA, RNA, and proteins were isolated from tumour and control tissues using the AllPrep DNA/RNA/Protein Mini Kit (Qiagen, Hilden, Germany), followed by an extension of the protocol to rescue the miRNAs, according to the manufacturer’s instructions. Total RNA was processed with RNase-Free DNase Set (Qiagen, Hilden, Germany) within the extraction protocol. We evaluated the quality and quantity of total RNA and miRNAs by microcapillary electrophoresis on the 2100 Bioanalyzer (Agilent Technologies, Santa Clara, CA, USA).

### 2.4. mRNA and miRNA Expression Analysis

Real time PCR (RT-PCR) was performed with the Revert Aid First Strand cDNA Synthesis Kit (Thermo Scientific, Waltham, MA, USA). To analyse miRNA expression, the RT-PCR was performed using the TaqMan MicroRNA Reverse Transcription Kit (Applied Biosystems, Foster City, CA, USA) with the RT-Primers pool, according to the manufacturer’s instructions.

The quantification of mRNA and microRNA expression was performed by quantitative real time PCR (qRT-PCR) with TaqMan technology. The TaqMan Gene Expression Assays and TaqMan MicroRNA Assays (Life Technologies, Carlsbad, CA, USA) chosen are detailed in Table 1. All analyses were done in duplicate and following the same protocol. Normal pituitary autopsy samples were mixed in equal parts into a pool that was analysed as a calibrator. The analyses were performed in 10-µL reaction volumes containing 5 µL of TaqMan Fast Advanced Master Mix (Life Technologies, Carlsbad, CA, USA), 0.5 µL of TaqMan Gene or miRNA Expression Assay (Life Technologies, Carlsbad, CA, USA), and 2.5 µL of template in the 7500 Fast Real Time PCR System (Applied Biosystems, Foster City, CA, USA), and were analysed using a SDS software (Applied Biosystems, Foster City, CA, USA). A relative quantification (RQ) was established based on the ΔΔCt method in FC units (Applied Biosystems, Foster City, CA, USA).

Gene and miRNA expression variables were categorised as repressed (RQ ≤ 0.5 FC), overexpressed (RQ ≥ 1.5 FC), or similar to physiological expression (normoexpressed: 0.5 FC < RQ < 1.5 FC) for statistical analysis.

### 2.5. Statistical Analysis

Molecular variables showed a non-normal distribution (Kolmogorov–Smirnov test). Associations between the molecular assays and the clinic-pathological features were calculated. Mann–Whitney U or Kruskal–Wallis tests were used for comparisons between two or more groups, as appropriate. The χ^2^ test followed by Fisher’s exact test when appropriate was used to identify the correlations between categorical variables. A non-parametric Spearman’s correlation test was used to test the association between molecular variables and age or MTD. The effect of a variable was estimated using 95% confidence interval (CI). For multivariable analysis, we used multiple linear regression between continuous variables and unconditional logistic regression between categorical variables. P values of less than 0.05 were considered statistically significant. Statistical analysis was performed with the SPSS 15.0 software (IBM Software, Armonk, NY, USA).

## 3. Results

We studied 60 samples from the collection of PitNETs of the biobank of the Alicante Health and Biomedical Research Institute. The demographic, clinical, and radiological characteristics of the patients are shown in Table 2.

### 3.1. Relationships between Genes (*E2F1/MYC*) and miRNAs Studied and PitNET Proliferation

**Figure 1:**
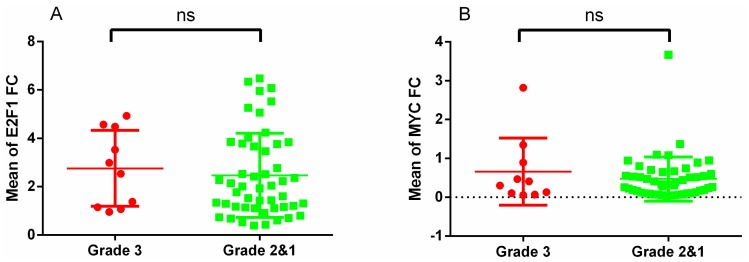
Association between E2F1 (**A**) and MYC (**B**) fold change expression and grades of invasiveness and proliferation (grade 3 vs. grade 2 and 1) in the whole series. Grade 3: proliferative and invasive tumours; grades 2 and 1: non-proliferative and non-invasive tumours. ns, non significant; FC, fold change.

There was no correlation in the expression of *E2F1* or *MYC* among the three categories of PitNETs in the whole series or by subtypes. When we grouped grades 1 and 2 (non-proliferative tumours) to compare them with grade 3 (proliferative tumours), the results did not change (Figure 1A,B).

**Figure 2:**
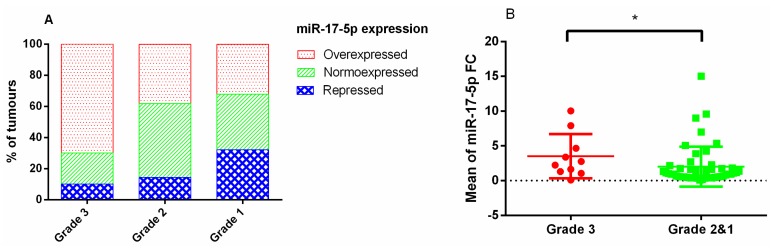
(**A**) Percentage of tumours with miR-17-5p overexpressed, normoexpressed, or repressed depending on grade of invasiveness and proliferation in the whole series; (**B**) Differences in miR-17-5p fold change expression between proliferative and invasive tumours (grade 3) and non-proliferative or non-invasive tumours (grades 2 and 1) in the whole series. * Mann–Whitney U, *p* = 0.041.

By contrast, we found significant differences in the expression of miR-17-5p in the whole series, when we compared grade 3 (proliferative tumours) vs. grades 2 and 1 (non-proliferative or non-invasive tumours) (Figure 2A,B). This difference was not observed in the subtypes.

Indeed, when we compared the expression of the previous genes and miR 17-5p in the tumours according only to their Ki-67 expression, only miR-17-5p was significantly higher in proliferative (Ki-67 ≥ 2.59) compared with non-proliferative tumours (Ki-67 < 2.59) (*p* = 0.05).

### 3.2. Relationships between Genes (*E2F1/MYC*) and miRNAs with the Invasiveness of the Tumours by Magnetic Resonance Imaging

To evaluate the association between the expressions of the genes and miRNAs studied with the invasiveness of PitNETs, we compared invasive tumours (grades 3 and 2) with non-invasive tumours (grade 1).

**Figure 3:**
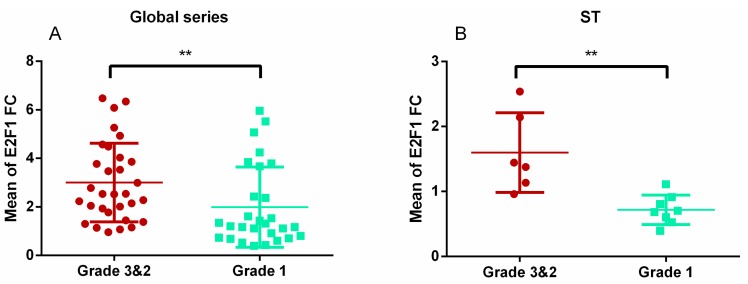
Different expression of E2F1 depending on invasiveness. E2F1 fold change values between invasive (grade 3 and 2) and non-invasive (grade 1) tumours in the whole sample (**A**) and in the ST subtype (**B**). ** Mann–Whitney U, *p* = 0.004 and *p* = 0.001, respectively).

**Figure 4:**
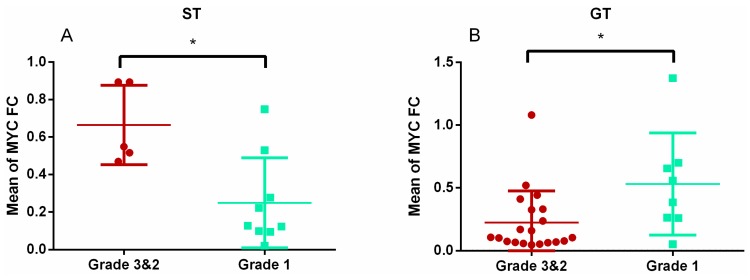
Different expression of MYC depending on invasiveness. MYC fold change values between invasive (grade 3 and 2) and non-invasive (grade 1) tumours in the ST (**A**) and in the GT subtypes (**B**). * Mann–Whitney U, *p* = 0.018 and *p* = 0.033, respectively.

In the whole series, invasive tumours showed higher *E2F1* gene expression than non-invasive ones (*p* = 0.004; Figure 3A). By subtypes, this finding was only maintained in STs (*p* = 0.001; Figure 3B). *MYC* also showed higher expression in invasive STs than in non-invasive ones (*p* = 0.018; Figure 4A), but not in the whole series. Indeed, the expression of *MYC* was lower in invasive than in non-invasive GTs (*p* = 0.033; Figure 4B).

Unlike *E2F1* and *MYC*, there were no differences in the expression of pri-miR-17-92 and mature miRNAs and invasiveness behaviour in the whole series or by subtypes.

### 3.3. Expression Profiles of *E2F1*, *MYC* Genes, and Pri-miR-17-92 by Subtypes

As shown in Table 3, *E2F1* was overexpressed in the silent (GTs and sCTs) and normoexpressed in functioning PitNET subtypes (fCTs and STs). Contrarily, *MYC* was normoexpressed only in the tumours of the corticotroph line (both functioning and silent corticotroph tumours) and repressed in the other subtypes (GTs and STs).

As shown in Table 4, Pri-miR-17-92 was repressed in all PitNET subtypes. However, mature miRNAs (miR-20a and miR-17-5p), similarly to *E2F1,* appeared slightly overexpressed in the silent subtypes (GT and sCT) and normoexpressed in functioning PitNET subtypes (fCTs and STs).

Finally, there was a negative correlation between *E2F1* and mature miRNAs expression in the whole series. Only pri-miR-17~92 correlated positively with *E2F1* in fCTs, but not in sCTs.

## 4. Discussion

In this paper, we try to find biomarkers of proliferation and invasiveness in PitNETs and report the possible implication of *E2F1*, *MYC*, pri-miR-17~92, miR-20a, and miR-17-5p in pituitary tumourigenesis. The strength of the paper is the size of the sample studied, which allows the analysis of different subtypes of PitNETs, and the scarcity of other published data. On the other hand, one limitation of the study is that we report correlations, which allow the generation of hypotheses, but not definitive conclusions on the relationship between *E2F1*, *MYC*, and pri-miR-17-92 in the tumourigenesis of PitNETs.

PitNETs are among the most common intracranial tumours and are usually benign neoplasms [21]. However, 30% to 40% of them invade surrounding structures, and relapses or re-growth during follow-up are frequent [22]. In the absence of more specific markers, IHC assessment of either p53 or the Ki-67 labelling index has been traditionally used as marker of aggressiveness. However, many researchers have raised doubts about their usefulness [12,23,24,25]. Indeed, some years ago, Trouillas et al. [19,20] suggested a classification of the behaviour of pituitary tumours taking into account not only markers of proliferation, but also the IHC type, the invasiveness of the tumours, and their ability to relapse. To the best of our knowledge, *E2F1* and *MYC* have never been studied as possible biomarkers of PitNETs.

*E2F1* is a transcription factor with activities related to either inhibiting or stimulating the development of the tumours; therefore, it could be considered a modulator of cell growth and death [1,2,3]. Recently, Fu et al. showed that *E2F1* was implicated in the overexpression of *CCAT2,* which, by interacting with *PPTG1,* could stimulate the development and progression of PitNETs [26]. In addition, Araki et al. also demonstrated that *E2F1* could play a role in pituitary tumourigenesis, through its induction of *POMC* and *ACTH* in fCT [27,28]. More recently, Metin-Armagan et al. have suggested the involvement of *E2F1* and other genes and proteins, such as *CHEK2* and p73, in the tumourigenesis process of non-functioning and functioning (Cushing Disease and Acromegaly) PitNETs [29]. Indeed, in the present study, we observed higher expression of *E2F1* in invasive versus non-invasive tumours in the whole series and in the ST subtype. However, we did not find differences in the expression of *E2F1* between proliferative (Ki-67 ≥ 2.59) and non-proliferative tumours. Thus, *E2F1* could be a potential biomarker of PitNET invasiveness, but not of proliferation.

*MYC* is an oncogene that participates in the tumourigenesis of most cancers and is associated with poor prognosis [30,31]. In PitNETs, Liu et al. found that the increase of *MYC* expression could predict the aggressiveness of silent tumours [32]. On the other hand, other authors found protein expression of *MYC* in just 27% of PitNETs [33]; thus, it can hardly be considered a biomarker of tumour behaviour. In our study, *MYC* was repressed in all PitNET subtypes, and there was no association between *MYC* and proliferation in the whole series or for subtypes. However, similarly to *E2F1*, we found a positive relationship between *MYC* expression and tumour invasiveness, although only in the ST subtype. The opposite occurred in the GT subtype. In conclusion, and like *E2F1*, *MYC* could not be considered a biomarker for PitNET proliferation. On the other hand, more studies are needed to establish a relationship between *MYC* expression and PitNET invasiveness, because its behaviour could depend on the tumour subtype.

miRNAs are a large group of small RNAs that do not code for proteins and are a fundamental component of gene regulation through the regulation of post-transcriptional events [34,35]. Di Ieva et al. have highlighted the possible role of miRNAs as circulating biomarkers of pituitary tumours, given the high vascularisation of the pituitary gland [11]. Moreover, a recent review performed by Wierinckx et al. highlights the relevant role of miRNAs in tumorigenesis and aggressiveness in PitNETs through their up- or down-regulation and their gene targets [36]. For instance, some years ago, down-regulation of a subset of miRNAs that target HMGA1 and HMGA2 (proteins overexpressed in malignant neoplasms [37]) were identified in PitNETs [38]. Similarly, D’Angelo et al. also observed a down-regulation of a set of miRNAs that target genes (such as *HMGA1, 2*, and *E2F1*) whose overexpression or activation plays a critical role in tumorigenesis in pituitary tumours of the somatotroph line [39,40]. Finally, the expressions of miR-23b and miR-130b, which target *HMGA2* and *CCNA2* genes [41], have been reported as down-regulated in somatotroph, gonadotroph, and silent PitNETs, while miR-183, which targets *KIAA0101* [42], appears to be down-regulated in aggressive lactotroph tumours.

In the present paper, we observed that miR-17-5p was overexpressed in proliferative tumours. Wei et al. [43] also have demonstrated higher miR-17-5p expression in pituitary carcinoma and atypical PitNETs compared with typical PitNETs. In their study, they suggest that miR-17-5p is involved in pituitary carcinoma metastasis, attenuating target genes such as *PTEN*, a tumour suppressor gene, and *TIMP2*, involved in inhibiting the degradation of the extracellular matrix and basement membrane. Therefore, miR-17-5p could be a good biomarker of proliferation in PitNETs.

Therefore, the higher expression of *E2F, MYC*, and miR-17-5p in invasive and proliferative tumours (grade 3) than in grade 1 (non-invasive tumours) confirms that both invasiveness and proliferation must be taken into account in PitNET classification [20]. In addition, the high expression of these genes and miRNA, implicated in the cancer, confirms that an invasive and proliferative tumour is not a benign adenoma as a non-invasive tumour, with or without proliferation [20].

In addition to the possible implication of *E2F1*, *MYC*, and miRNAs in the behaviour of PitNETs, they could also participate in their functionality. However, there is very little information in the literature. Only Tani et al. looked for differences in the expression of *E2F1* between functioning and silent subtypes, without finding any changes [44]. Contrarily, we found that *E2F1* was overexpressed in the silent subtypes (GTs and sCTs) and normoexpressed in functioning PitNET subtypes (fCTs and STs). Nonetheless, functioning PitNETs comprised both macroadenomas and microadenomas, whereas all operated silent tumours were macroadenomas. Therefore, there is an important bias of size when trying to interpret the role of *E2F1* in the functionality of PitNETs.

**Figure 5:**
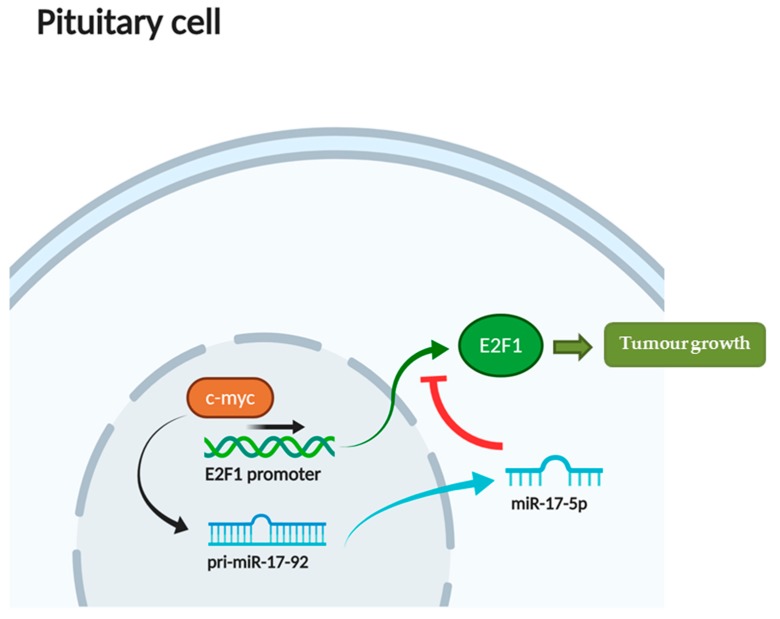
Participation of mature miR-17-5p in the regulation of *MYC* and *E2F1* networks in PitNETs.

As previously noted, *E2F1* is under the transcriptional regulation of *MYC* [7] and of some miRNAs. In the present paper, *E2F1* was overexpressed while *MYC* was repressed in GTs, which suggests a negative feedback between *E2F1* and *MYC* in the regulation of cellular cycle in this subtype. The negative feedback between *E2F1* and *MYC* has been previously described by Zhang et al. in human oesophageal fibroblast (HOF) cells [45,46], through the negative effect of *E2F1* on hTERT transcription [45]. However, positive feedback between *E2F1* and *MYC* has also been reported [47,48]. Moreover, it has been recently suggested that the miR-17~92 cluster also participates in the regulation of *MYC* and *E2F1* networks [14,49,50,51]. Indeed, in our study, miR-17-5p was also overexpressed in GTs. The possible interrelations among *E2F1, MYC*, and miRNAs are outlined in Figure 5, which shows that the negative effect of *MYC* on *E2F1* translation, mediated by miR 17-5p, would surpass its positive effect on *E2F1* transcription. A similar relationship between *MYC*/*E2F1* and miR-17-5p was observed in sCTs, but not in the functioning variants of PitNETs (fCTs and STs.) As all non-functioning PitNETs (GTs and sCTs) were macroadenomas, while some functioning PitNETs (fCTs and STs) were microadenomas, the effect of *E2F1* on the growth and invasiveness of pituitary tumours could be mediated by its interaction with *MYC* and miR-17-5p.

## 5. Conclusions

Our study suggests that in PitNETs, *E2F1* could be a good biomarker of invasiveness, and miR-17-5p of proliferation, helping the clinical management of these tumours. In contrast, *MYC*’s role in PitNET behaviour could be subtype-dependent. The expression of these genes must be evaluated in recurrent/progressive or aggressive PitNETs to precise if they are prognostic markers of tumour behaviour.

Despite some differences in *E2F1* expression between functioning (ST and fCT) and non-functioning (GT and sCT) PitNETs, it is difficult to establish a relationship between *E2F1* expression and functionality, because all silent operated tumours were macroadenomas, while most STs and fCTs were microadenomas. Finally, the effect of *E2F1* on the growth of PitNETs could be mediated by a complex interaction between *MYC* and miR-17-5p.

## Figures and Tables

**Table 1:** TaqMan Expression Assays chosen (Life Technologies).

Gene		TaqMan Gene Expression Assays
	*E2F1*	Hs00153451_m1
	*MYC*	Hs00153408_m1
	*Ki-67*	Hs01032443_m1
	Pri-miR~17-92	Hs03295901_pri
Housekeeping genes		
	*ACTB*	Hs99999903_m1
	*GAPDH*	Hs99999905_m1
	*YWHAZ*	Hs00237047_m1
**microRNA**		**TaqMan miRNA Expression Assays**
	Hsa-miR-17-5p	002308
	Hsa-miR-20a	000580
Small nucleoar RNA (for normalisation)		
	RNU43	001095
	U54	001210

**Table 2:** Baseline characteristics of included patients with pituitary neuroendocrine tumours (PitNETs) (*N* = 60).

Variables			Frequency or Mean ± SD
Age (years)			54 ± 15
Women			33/60 (55%)
Maximum tumour diameter (mm)			22.51 ± 10.92
PitNET subtypes			
	ST tumours		15/60 (25%)
	fCT tumours		8/60 (13.33%)
	sCT tumours		8/60 (13.33%)
	GT tumours		29/60 (48.33%)
Invasiveness		Knosp grade	59/60 *
	Non-invasive	Grades I–II	28/59 (47.5%)
	Invasive	Grades III–IV	31/59 (52.5%)
Proliferation		Ki-67	59/60 *
	Non-proliferative	<2.59	42/59 (71.2%)
	Proliferative	≥2.59	17/59 (28.8%)
Grades			59/60 *
	Grade 1	Non-invasive (regardless Ki-67 index)	28/59 (47.5%)
	Grade 2	Invasive and non-proliferative (Ki-67 < 2.59)	21/59 (35.6%)
	Grade 3	Invasive and proliferative (Ki-67 ≥ 2.59)	10/59 (16.9%)

SD: standard deviation; ST: functioning somatotroph; fCT: functioning corticotroph; sCT: silent corticotroph; GT: gonadotroph * One case missed.

**Table 3:** Distribution of *E2F1* and *MYC* expressions (fold change) according to PitNET subtype.

Subtype	*E2F1*	*MYC*
	Mean Fold Change (IQR)	
All	2.09 (1.16–3.78)	0.32 (0.11–0.64)
GT	3.03 (2.01–4.03) *	0.17 (0.07–0.41) ^†^
fCT	1.32 (1.17–1.90) *	0.59 (0.44–1.02) ^†^
sCT	3.45 (1.01–5.00)	0.70 (0.25–1.88) ^†^
ST	0.96 (0.69–1.41) *	0.28 (0.13–0.54) ^†^
*p* value	<0.001	0.004

GT: gonadotroph; fCT: functioning corticotroph, IQR: interquartile range, sCT: silent corticotroph, ST: functioning somatotroph). *p* values: according to Kruskal–Wallis test for differences between PitNET subtypes. * Differences between GT and fCT (*p* = 0.002) and between GT and ST (*p* < 0.001). ^†^ Differences between fCT and ST (*p* = 0.047), between fCT and GT (*p* = 0.002), between ST and sCT (*p* = 0.056), and between sCT and GT (*p* = 0.009).

**Table 4:** Distribution of Pri-miR-17~92 and mature microRNAs expression (fold change) according to PitNET subtype.

Subtype	Pri-miR-17~92	miR-17-5p	miR-20a
	Mean Fold Change (IQR)		
All	0.12 (0.08–0.27)	1.15 (0.53–2.61)	1.21 (0.51–2.54)
GT	0.11 (0.07–0.25)	1.62 (0.92–2.77)	1.69 (0.80–2.82)
fCT	0.13 (0.09–0.24)	0.69 (0.53–1.27)	1.02 (0.53–1.28)
sCT	0.22 (0.13–0.31)	1.59 (0.54–6.84)	1.56 (0.55–8.44)
ST	0.12 (0.09–0.42)	0.92 (0.42–1.56)	0.83 (0.46–1.93)
*p* value	0.421	0.286	0.477

GT: gonadotroph; fCT: functioning corticotroph; IQR: interquartile range; sCT: silent corticotroph; ST: somatotroph. *p* values: according to Kruskal–Wallis test for differences between PitNET subtypes.

## Abbreviations

CI	Confidence interval
CT	Corticotroph tumour
FC	Fold change
fCT	Functioning corticotroph tumour
GT	Gonadotroph
HOF	Human oesophageal fibroblast
IHC	Immunohistochemical
IQR	Interquartile range
MTD	Maximum tumour diameter
MRI	Magnetic resonance image
OR	Odds ratio
qRT-PCR	Quantitative real time polymerase chain reaction
ROC	Receiver operator curve
RQ	Relative quantification
RT-PCR	Real time polymerase chain reaction
sCT	Silent corticotroph tumour
ST	Somatotroph tumour
